# Investigation of a complete squeeze-film damping model for MEMS devices

**DOI:** 10.1038/s41378-021-00279-6

**Published:** 2021-07-22

**Authors:** Qianbo Lu, Weidong Fang, Chen Wang, Jian Bai, Yuan Yao, Jiaxiao Chen, Xiang Xu, Wei Huang

**Affiliations:** 1 Ningbo Institute of Northwestern Polytechnical University, Frontiers Science Center for Flexible Electronics (FSCFE), MIIT Key Laboratory of Flexible Electronics (KLoFE), Shaanxi Key Laboratory of Flexible Electronics (KLoFE), Institute of Flexible Electronics (IFE), Northwestern Polytechnical University, 710072 Xi’an, Shaanxi China; 2 College of Optical Science and Engineering, State Key Laboratory of Modern Optical Instrumentation, Zhejiang University, 310027 Hangzhou, China; 3 Department of Electrical Engineering and Computer Science, University of Liege, Liege, Belgium; 4 ESAT-MNS, University of Leuven, 3001 Leuven, Belgium; 5 Huazhong University of Science and Technology - Wuhan National Laboratory for Optoelectronics, 430074 Hubei, China; 6 Huazhong Institute of Electro-Optics - Wuhan National Lab for Optoelectronics, 430074 Hubei, China

**Keywords:** Sensors, NEMS

## Abstract

Dynamic performance has long been critical for micro-electro-mechanical system (MEMS) devices and is significantly affected by damping. Different structural vibration conditions lead to different damping effects, including border and amplitude effects, which represent the effect of gas flowing around a complicated boundary of a moving plate and the effect of a large vibration amplitude, respectively. Conventional models still lack a complete understanding of damping and cannot offer a reasonably good estimate of the damping coefficient for a case with both effects. Expensive efforts have been undertaken to consider these two effects, yet a complete model has remained elusive. This paper investigates the dynamic performance of vibrated structures via theoretical and numerical methods simultaneously, establishing a complete model in consideration of both effects in which the analytical expression is given, and demonstrates a deviation of at least threefold lower than current studies by simulation and experimental results. This complete model is proven to successfully characterize the squeeze-film damping and dynamic performance of oscillators under comprehensive conditions. Moreover, a series of simulation models with different dimensions and vibration statuses are introduced to obtain a quick-calculating factor of the damping coefficient, thus offering a previously unattainable damping design guide for MEMS devices.

## Introduction

Dynamic performance, which is of paramount importance for micro-electro-mechanical system (MEMS) devices, is bounded by the surface forces, also known as damping^1–3^. For example, the bandwidth and frequency response of a MEMS accelerometer^4^, the mechanical response of a resonator^5^, and the contact times of a switch^6^, shock-absorbent squeeze-film damper^7,8^ and other nonvacuum vibration transducers^9,10^ are all substantially affected by damping. Therefore, understanding the mechanism that contributes to air damping carries great significance for the MEMS community, because it can offer guiding rules for MEMS design and can enable the dynamic performance tuning of various kinds of MEMS devices^11^.

Air damping can be categorized as squeeze-film air damping or slide-film air damping, which denote the air reaction, while two parallel plates move toward each other and in parallel, respectively. Squeeze-film air damping is dominant in MEMS devices, and the influence becomes significant with decreasing device dimensions. The air film can be perceived as a combination of a spring and a damper during squeeze-film air damping analysis^12^, whereas the key figure of merit becomes the calculation of the air damping coefficient. Tipei^1^ introduced the Reynolds equation half a century ago, which is most widely used in the investigation of squeeze-film air damping based upon Reynolds’ research^13^. Thereafter, a series of solutions for the damping coefficient in different cases were proposed^12,14–16^, although most of them focused on cases of small-amplitude vibration with trivial boundary conditions.

However, combining the border effect along with the amplitude effect may cause a deviation of >35% compared with cases of small-amplitude vibration with trivial boundary conditions^17^. Expensive efforts have been undertaken to take these two effects into account. For example, regarding the border effect, Veijola et al.^18,19^ proposed an assumption of air flow channels to characterize the border effect and built an elongation model for rectangular plates, whose accuracy was higher than that of the simplest model, while estimating the damping coefficient of small-amplitude vibration structures. For the amplitude effect, Sadd and Stiffer^20^ opened a path toward the solution of squeeze-film air damping with amplitude effects and proposed formulas for large-amplitude damping coefficients, but their theory only applied to the trivial boundary condition. In addition, many studies have focused on the impact of large amplitudes, but no border effect consideration has been included simultaneously, leading to a lack of accurate estimates^21–23^. In summary, investigations considering both the border effect and amplitude effect have remained elusive, which is the subject of this paper, and essential for the MEMS community because MEMS devices in reality can have complicated boundary conditions, and the vibration amplitude can even exceed 50% of the air film thickness.

In this paper, a more complete squeeze-film air damping model is proposed that considers both the border effect and amplitude effect. We first discuss the simplest model of squeeze-film air damping, and then modify the model by adding the border effect for cases of rectangular and circular plates. Thereafter, we present a complete theoretical model, wherein the model considering the border effect and amplitude effect together gives the analytical expression of the damping coefficient of the squeeze film. A series of simulation models are built to compare the theoretical model and simulation results in different cases, including cases with border effect only, amplitude effect only, and both effects, which in turn verifies the consistency and reliability of the analytical expression. Experimental measurements based on the free-vibration decay (FVD) method further confirm the validity of the theoretical model and simulation. In addition, the damping model is further expanded on the basis of the simulation results to obtain a general expression containing a quick-calculating factor, which can be considered a quick and strong guide in the damping-related analysis and corresponding MEMS design.

## Results

### Complete squeeze-film air damping model

#### Simplest model of squeeze-film air damping without border effect and amplitude effect

Squeeze-film air damping represents the effect of the opposite force of air on the movable structures, when the air is squeezed or sucked. The theory of squeeze-film air damping has received extensive attention over the past decades. In this section, we first give the simplest case of squeeze-film air damping without the border effect and amplitude effect.

In a typical case of squeeze-film air damping, the moving direction of a parallel plate is perpendicular to the wall, and air between them leaves laterally. Considering the conservation of air mass, the general Reynolds equation gives the dynamic performance of the air film between the wall and the plate^24^:

1 $$\frac{\partial }{{\partial X}}\left( {\rho \frac{{H^3}}{\mu }\frac{{\partial P}}{{\partial X}}} \right) + \frac{\partial }{{\partial Y}}\left( {\rho \frac{{H^3}}{\mu }\frac{{\partial P}}{{\partial Y}}} \right) = 12\left( {\frac{{\partial (H\rho )}}{{\partial T}}} \right),$$

where *P* is the pressure distribution function of the air film, and the variation in pressure along the moving direction of the plate can be ignored due to the small thickness, *ρ* is the density of the air film, *H* is the thickness of the air film, *μ* is the coefficient of viscosity of the air film, *X* and *Y* are coordinates, and *T* represents the time. The general Reynolds equation is applicable to any shape plate, including a rectangular plate and circular plate.

On the basis of Eq. (1), for an oscillating plate, *H* can be defined as:

2 $$H = H_0\left( {1 + \varepsilon \sin \left( {\omega T} \right)} \right),$$

*H*_0_ is the initial thickness of the air film, *ε* is the amplitude ratio, which equals the ratio of the oscillation amplitude of the plate to the initial thickness of the air film, and *ω* is the frequency of movement. In addition, the air density *ρ* can be replaced by the pressure *P* relying on the assumption of consistent temperature in MEMS devices. For further simplification, the parameters are first normalized to obtain the simplified form of the Reynolds equation as:

3 $$\frac{{\partial ^2p^2}}{{\partial x^2}} + \frac{{\partial ^2p^2}}{{\partial y^2}} = \frac{{2\sigma }}{{h^3}}\frac{{\partial (hp)}}{{\partial t}},$$

where *p* = *P/P*_0_, *x* = *X/l*, *y* = *Y/l*, *h* = *H/H*_0_, *t* = *ωT*, *P*_0_ is the ambient pressure, and *l* is the characteristic length of the plate (equal to half the width of the rectangular plate *W* or the radius of the circular plate *R*). In addition, the squeeze number *σ*, which is utilized in the following analysis, has the following definition:

4 $$\sigma = \frac{{12\mu \omega l^2}}{{H_0^2P_0}}.$$

For the simplest case, in which the oscillation amplitude of the plate is small (*H* is approximately equal to *H*_0_) and the pressure is zero at the plate boundary, the damping coefficient of parallel plates can be calculated as^25^:

5 $$c_{\mathrm{{d - sp}}} = f\left( \gamma \right)\frac{{\mu l^4}}{{H_0^3}},$$

The factor *f* (*γ*) for the rectangular plate and circular plate can be respectively represented as:

6 $$\left\{\begin{array}{lll}f_{\mathrm{rec}}\left(\gamma\right)= 16\gamma\left({1 -\frac{{192\gamma}}{{\pi ^5}}\mathop {\sum}\limits_{n=\mathrm{odd}} {\frac{1}{n ^5}\tanh \frac{{n\pi}}{{2\gamma}}}}\right)\\ f_{\mathrm{cyc}}\left(\gamma\right)=\frac{{3\pi}}{2}\end{array}\right.,$$

where *γ* denotes the ratio of width and length, ranging from 0 to 1 for a rectangular plate and 1 for a circular plate.

The general Reynolds equation has served as a theoretical underpinning for damping issues over the past half-century. Researchers took full advantage of this simplest model to obtain the damping coefficient for different types of squeeze-film air damping, whereas they corresponded to various assumptions.

However, the simplest model is only applicable to small-amplitude and trivial boundary conditions with compressible air, which has heavy limitations when facing complicated conditions. For example, the nonlinearity due to a rotating or a large-amplitude movement of the plate is a challenging problem. In addition, different boundary conditions, in terms of different geometries, are a difficult to establish using the basic theory. Some researchers, such as Veijola^18,19^ and Sadd^20^, have developed simple approaches to calculate squeeze-film air damping involving complicated boundary conditions and large-amplitude vibrations, which are termed the border effect and amplitude effect, respectively. Nevertheless, conventional theories have not considered them together, so that there always exists a nonnegligible deviation from reality.

In the next sections, we further develop the model, inspired by previous scholars’ work, and propose a complete model to evaluate the dynamic characteristics of air damping by considering both the border effect and amplitude effect for universal structures.

#### Modified model of squeeze-film air damping with border effect only

**Fig. 1:**
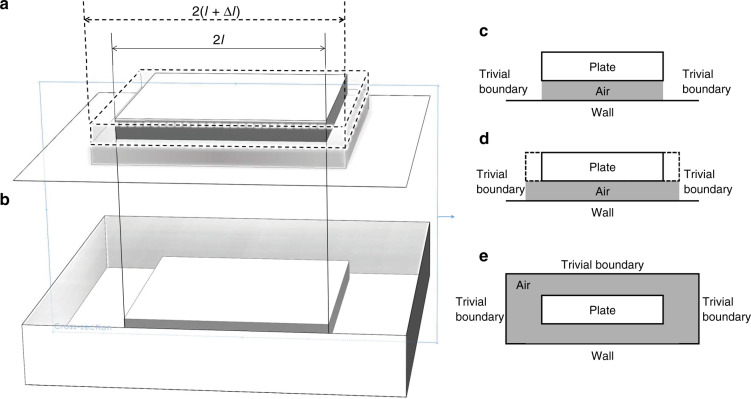
Schematic of boundary conditions. **a** simplest model (solid line) and elongation model (dashed line) with trivial boundary conditions. **b** Complete model with border effects. **c**, **d** and **e** Cross-sectional views of these three models.

The aforementioned solution is based on the trivial boundary condition, in which the relative pressure around the plate boundary is regarded as zero, as shown in Fig. 1a, c. However, for a complete model of squeeze-film air damping, as shown in Fig. 1b, e, the pressure around the plate is nonzero, and the moving air undoubtedly has effects on the plate.

Inspired by previous research^18,19^, the movement condition of air sandwiched between the plate and wall in flow channels with dimensions of 2*W* × 2*L* × *H* for a rectangular plate or 2*R* × Δ*w* × *H* for a circular plate with border effects is considered to be equivalent to the air flow in elongated channels without border flow, wherein elongated channels have an elongated width Δ*W* and length Δ*L* for a rectangular plate and an elongated radius Δ*R* for a circular plate.

The modified elongation model is shown in Fig. 1a, d with dashed lines, which have been proven to successfully approximate the real boundary condition for squeeze-film air damping. The dimensional parameters of the elongation model, which are termed *W*_elong_ = *W* + Δ*W*, *L*_elong_ = *L* + Δ*L*, and *R*_elong_ = *R* + Δ*R*, are calculated as^26^:

7 $$\left\{ \begin{array}{l}W_{\mathrm{elong}} = W\frac{{\sqrt {1 + 3A_W} \left( {1 + 4A_L} \right)^{3/8}}}{{\sqrt {1 + 3A_L} \left( {1 + 4A_W} \right)^{1/8}}}\\ L_{\mathrm{elong}} = L\frac{{\sqrt {1 + 3A_L} \left( {1 + 4A_W} \right)^{3/8}}}{{\sqrt {1 + 3A_W} \left( {1 + 4A_L} \right)^{1/8}}}\end{array} \right.,$$

8 $$\left\{ \begin{array}{l}A_W = \frac{4}{{3\pi }}\frac{{1 + 2.676K_n^{0.659}}}{{1 + 0.531K_n^{0.5}\left( {H/2W} \right)^{0.238}}}\frac{H}{W}\\ A_L = \frac{4}{{3\pi }}\frac{{1 + 2.676K_n^{0.659}}}{{1 + 0.531K_n^{0.5}\left( {H/2L} \right)^{0.238}}}\frac{H}{L}\end{array} \right.,$$

9 $$\left\{ \begin{array}{l}R_{\mathrm{elong}} = R\frac{{\left( {1 + 3A_R} \right)^{1/2}}}{{\left( {1 + 4A_R} \right)^{1/8}}}\\ A_R = \frac{4}{{3\pi }}\frac{{1 + 2.676K_n^{0.659}}}{{1 + 0.531K_n^{0.5}(H/2R)^{0.238}}}\frac{H}{R}\end{array} \right..$$

where *K*_*n*_ is the Knudsen number, which is close to zero when the air film thickness is large, and Δ*w* is set close to zero to simplify the expression of the elongated radius of the circular plate. Similar formulas are obtained by FEM simulation as well^17^. When the dimensions of the plates are larger than the thickness of the air film, the first-order approximation of the elongation dimensions can be expressed as:

10 $$\left\{ \begin{array}{l}W_{\mathrm{elong}} \approx W\left( {1 + A_W} \right) \approx W\left( {1 + \frac{4}{{3\pi }}\frac{H}{W}} \right)\\ L_{\mathrm{elong}} \approx L\left( {1 + A_L} \right) \approx L\left( {1 + \frac{4}{{3\pi }}\frac{H}{L}} \right)\\ R_{\mathrm{elong}} \approx R\left( {1 + A_R} \right) \approx R\left( {1 + \frac{4}{{3\pi }}\frac{H}{R}} \right)\end{array} \right..$$

By introducing the characteristic length *l*, Eq. (10) can be simplified to:

11 $$l_{\mathrm{elong}} = l\left( {1 + \frac{4}{{3\pi }}\frac{H}{l}} \right).$$

Substituting the elongated dimensional parameter into Eqs. (5) and (6), we therefore obtain the squeeze-film air damping coefficient with the border effect of the elongation model:

12 $$c_{\mathrm{{d - be}}} = f\left( \gamma \right)\frac{{\mu l^4}}{{H_0^3}} \times \left( {1 + \beta } \right)^4,$$

where *β* is a factor of border effect that equals *4H*_0_*/*3*πl*. Due to the small value of *β* in most cases, *γ* can be considered a constant in the elongation model.

#### Complete model with border effect and amplitude effect

While the oscillation amplitude of the plate is large enough, for example, comparable to the air film thickness, it can lead to a quite different dynamic damping effect compared to the small-amplitude case, termed the amplitude effect. To obtain a more applicable theoretical expression, taking the large oscillation amplitude condition into account, we raise the presumption that the squeeze number *σ* is small, which means that air is incompressible or has enough time to leak from the gap. In addition, the dimensions of the plate should be far larger than the thickness of the air film. On the basis of this assumption and Eq. (3), it is straightforward to obtain a solution for rectangular and circular plates with trivial boundary conditions and amplitude effects^20^, which combines the amplitude effect function^25^ and equation of the simplest model to successfully predict the dynamic performance of squeeze-film air damping with a large-amplitude oscillation and trivial boundary condition:

13 $$c_{\mathrm{{d - ae}}} = f\left( \gamma \right)\frac{{\mu l^4}}{{H_0^3}} \times \frac{1}{{\left( {1 - \varepsilon ^2} \right)^{1.5}}}.$$

We then combine the work of the amplitude effect and border effect together, further developing an elongation model in consideration of the amplitude effect, which is termed the complete model. Regarding the elongation dimensions mentioned in Eq. (11), we obtain the elongation boundary conditions of the rectangular and circular plates:

14 $$\left\{ {\begin{array}{*{20}{l}} {p( \pm l_{\mathrm{elong}},y,t) = 0} \hfill \\ {p\left( {x, \pm \frac{{l_{\mathrm{elong}}}}{\gamma },t} \right) = 0} \hfill \end{array}} \right.$$

and

15 $$p\left( {l_{\mathrm{elong}},\theta ,t} \right) = 0.$$

The modified normalized Reynolds equation is then obtained by introducing two normalized parameters $\tilde x = X/l_{\mathrm{elong}}$ and $\tilde y = Y/l_{\mathrm{elong}}$:

16 $$\frac{{\partial ^2p^2}}{{\partial \widetilde x^2}} + \frac{{\partial ^2p^2}}{{\partial \widetilde y^2}} = \frac{{2\sigma }}{{h^3}}\left( {1 + \beta h} \right)^2\frac{{\partial \left( {hp} \right)}}{{\partial t}}.$$

The squeeze number *σ* is assumed to be small so that the pressure *p* can be Taylor expanded:

17 $$p = 1 + p_1\sigma + p_2\sigma ^2 + 0(\sigma ^3).$$

Substituting Eq. (17) into Eq. (16) and combining the boundary conditions in Eqs. (14) and (15), the time-varying damping forces on the vibrated rectangular plate and circular plate are obtained, respectively:

18 $$\begin{array}{ll}F\left( t \right) &= \,4\gamma P_0l^2\left( {1 + \beta h} \right)^3\left( {1 + \beta h} \right)\left[ { - \frac{{2h^{\prime} \sigma }}{{h^3}}\mathop {\sum}\limits_{n = \mathrm{odd}} {\left( {\frac{2}{{n\pi }}} \right)^4\left( {1 - \frac{{\tanh \frac{{n\pi }}{{2\gamma }}}}{{\frac{{n\pi }}{{2\gamma }}}}} \right)}} \right.\\ &\quad\left.+ \,{\left( {\frac{{h^{\prime\prime} }}{{h^5}} - \frac{{5h^\prime }}{{2h^6}}} \right)\sigma ^2\mathop {\sum}\limits_{n = \mathrm{odd}} {{\sum} {\left( {\frac{2}{{n\pi }}} \right)^6} \left[ {3\left( {1 - \frac{{\tanh \frac{{n\pi }}{{2\gamma }}}}{{\frac{{n\pi }}{{2\gamma }}}}} \right) - \tanh ^2\frac{{n\pi }}{{2\gamma }}} \right]} } \right]\end{array},$$

and

19 $$F\left( t \right) = 4\pi P_0l^2\left( {1 + \beta h} \right)^4\left[ { - \frac{1}{8}\frac{{h^\prime }}{{h^3}}\sigma + \frac{1}{{48}}\left( {\frac{{h^{\prime\prime} }}{{h^5}} - \frac{{5\left( {h^\prime } \right)^2}}{{2h^6}}} \right)\sigma ^2} \right].$$

The damping force has the following definition:

20 $$F\left( t \right) = kH + cH^\prime,$$

where *k* and *c* denote the stiffness coefficient and damping coefficient, respectively. In addition, the damping force on the plate can be expanded to a Fourier series, expressed as follows:

21 $$\begin{array}{l}F\left( t \right) =A_0 + A_1\cos t + B_1\sin t \\\quad\qquad \,+ \cdots + A_n\cos (nt) + B_n\sin (nt).\end{array}$$

From the previous two equations, the modified stiffness coefficient and damping coefficient can be derived, and in turn, the damping coefficient of the time-varying elongation model of the rectangular plate and circular plate are expressed as:

22 $$c_{\mathrm{{d - ab}}} = f\left( \gamma \right)\frac{{\mu l^4}}{{H_0^3}} \times g\left( {\varepsilon ,\beta ,\gamma } \right),$$

23 $$\begin{array}{ll}g\left( {\varepsilon ,\beta ,\gamma } \right) = \left[ {\frac{1}{{\left( {1 - \varepsilon ^2} \right)^{1.5}}} + \left( {3 + \gamma } \right)\beta \frac{{2\left( {1 - \sqrt {1 - \varepsilon ^2} } \right)}}{{\varepsilon ^2\sqrt {1 - \varepsilon ^2} }}}\right.\\ \qquad\quad\qquad\left.{+ \,3\left( {1 + \gamma } \right)\beta ^2\frac{{2\left( {1 - \sqrt {1 - \varepsilon ^2} } \right)}}{{\varepsilon ^2}} + \left( {3\gamma + 1} \right)\beta ^3 + \gamma \beta ^4} \right].\end{array}$$

Equation (22) can be perceived as a more trustworthy estimation of the damping coefficient by considering both the border effect and amplitude effect, which we believe can be a more applicable guide in MEMS design.

### Verification and development of the complete model

#### Simulation verification

Numerical methods have been proven to deal with the issue of fluid dynamic performance with high accuracy, and have effectively simulated the influence of air around a plate boundary, but at the cost of computational overhead and time, especially for complicated structures.

**Fig. 2:**
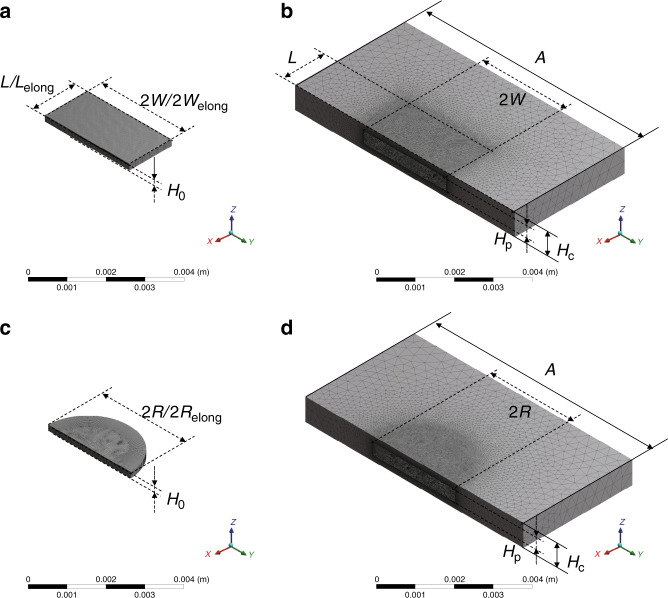
Meshed diagrams overview. **a** simplest model or elongation model for rectangular plate. **b** complete model for rectangular plate. **c** simplest model or elongation model for circular plate. **d** complete model for circular plate.

In this section, the correctness of the complete theoretical model is verified by using self-built simulation models. Herein, four simulation models are introduced, and the meshed diagrams are shown in Fig. 2, which are the simplest models (the same as the elongation models) with trivial boundary conditions and complete models for rectangular plates (Fig. 2a, b) and circular plates (Fig. 2c, d), respectively. Note that the only difference between the simplest models and the elongation models is the dimensions of the plate; thus, it can be analyzed by the same simulation model. The detailed parameters are listed in Table 1, and the lateral displacement of the plate is set to zero in simulations, indicated by *x* *=* *y* = 0. The displacement of the plate along the *z*-axis is defined as:

24 $$z = \varepsilon \sin \left( {\omega T} \right).$$

**Table 1:** Initial parameters of the elongation models.

Geometry parameters	
Half width of rectangular plate, *W* (μm)	1500
Half length of rectangular plate, *L* (μm)	1500
Radius of circular plate, *R* (μm)	1500
Calculated elongation width, *W*_elong_ (μm)	1579
Calculated elongation length, *L*_elong_ (μm)	1579
Calculated elongation radius, *R*_elong_ (μm)	1581
Thickness of air film, *H*_0_ (μm)	200
Thickness of plate, *H*_p_ (μm)	400
Thickness of complete model, *H*_c_ (μm)	800
Oscillation frequency of plate, *ω* (rad/s)	2*π* × 100
Fluid properties, air	
Ambient pressure, *P*_0_ (Pa)	1.01 × 10^5^
Temperature, *T* (K)	300
Viscosity coefficient of air, *μ* (N × s)/m^2^	1.7984 × 10^−6^

The damping coefficients are calculated based on the pressure distribution of the air film extracted from the simulation results^27^:

25 $$c_{\mathrm{d}} = {\mathrm{Re}} \left( {\frac{{{\int} {pdA} }}{{H^\prime }}} \right).$$

Simulations were conducted under different conditions, including considering only the border effect, only the amplitude effect, and both with different structural dimensions.

**Fig. 3:**
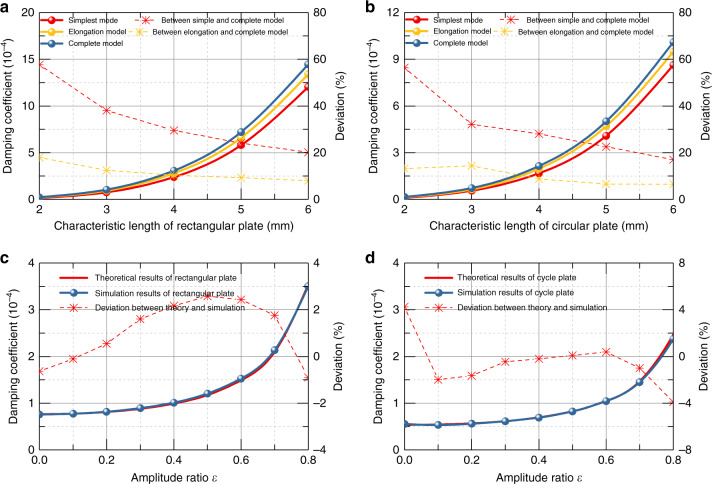
Simulation results of damping coefficient and deviation versus different dimensions of different models. **a** Results of damping coefficient versus characteristic length for rectangular plate with small amplitude. **b** Results of damping coefficient versus characteristic length for circular plate with small amplitude. **c** Results for rectangle plate with amplitude effect only. **d** Results for circular plate with amplitude effect only.

First, we utilized the simulation models of the simplest model, elongation model (the same form as the simplest model but different dimensions) and complete model, as shown in Fig. 2, to verify the case with border effects only, while the dimension of the elongation model was obtained from Eq. (11). The characteristic length of both rectangular and circular plates was altered from 2 to 6 mm in the simulation. Figure 3a, b shows the simulated damping coefficient with small amplitudes and deviations of rectangular and circular plates, respectively, in which the solid circles represent the damping coefficient and stars represent the relative deviation from the results of the complete model. The deviations obviously show that the elongation model results appear to be much closer to the complete results compared with the simplest model. While the deviations increase with the decrease in the characteristic length due to the size effect, the deviations between the elongation model and the complete model are always three times smaller than those of the simplest model, which confirms the validation of the elongation models for cases with border effects only. We then compared the theoretical and simulation results for the case with the amplitude effect, but without the border effect. The simulation models are shown in Fig. 2a, c, and the theoretical result is based on Eq. (13). The simulation was performed by setting the amplitude ratio *ε* from 0 to 0.8 for the rectangular plate and circular plate. The relative deviation of the damping coefficient can be clearly found to be small, which is presented in Fig. 3c, d. It is noted that the deviations of the damping coefficient are all <5%. The comparison indicates that the theoretical and simulation results have high accordance in the case with the amplitude effect, but without the border effect.

**Fig. 4:**
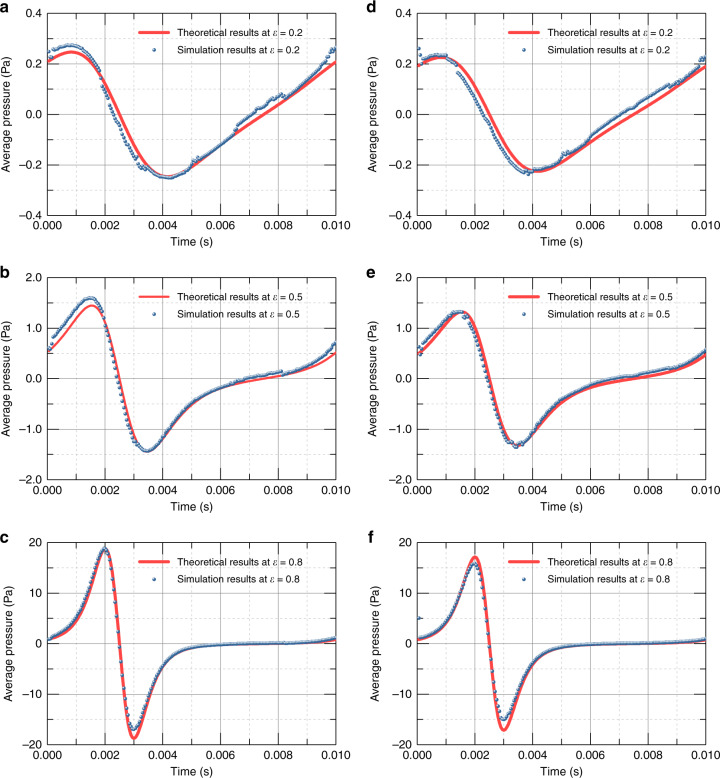
Theoretical and simulated average damping pressure. **a–c** Theoretical and simulated average damping pressure loading on a rectangular plate. **d**–**f** Theoretical and simulated average damping pressure loading on a circular plate for the complete model.

The complete models with border effects and amplitude effects, as shown in Fig. 2b, d, were then simulated with different amplitude ratios *ε* in comparison with the theoretical results of the complete model. Figure 4 shows the pressure of the theoretical and simulation results with various times. It is demonstrated that the complete model in consideration of both effects, represented by the red curve, better confirms the simulation results, which serve as the baseline of the true result.

**Fig. 5:**
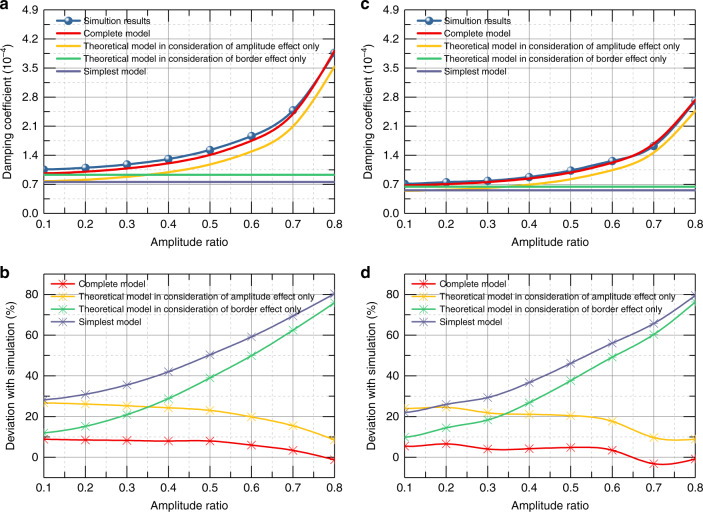
Damping coefficient of simulation results and theoretical results. **a**–**b** Damping coefficient along with the deviation of simulation results and theoretical results of complete model, in consideration of amplitude effect only, in consideration border effect only and simplest model versus amplitude ratio for rectangle plate. **c**–**d** Damping coefficient along with the deviation of simulation results and theoretical results of complete model, in consideration of amplitude effect only, in consideration border effect only and simplest model versus amplitude ratio for circular plate.

Moreover, Fig. 5 shows the results of all cases, including the simplest model, modified theoretical model with border effect only, model with amplitude effect only, and the complete model. It is obvious that the deviation relative to the simulation results decreases dramatically from the simplest model to the complete model, from nearly 80% for the simplest model to <9% for the complete model, which is a powerful confirmation of the effectiveness of our proposed model. Even when compared with the up-to-date theoretical model, the complete model shows a threefold improvement. It is likewise interesting to observe the decrease in the relative deviation of the theoretical result of models in consideration of the amplitude effect, with the increase in the amplitude ratio. The reason could be that the ratio of the air film thickness to the characteristic length of the plate, indicated by *H*_0_/*l*, decreases with increasing amplitude ratio, which then leads to the weakening of the border effect.

#### Experimental verification

To experimentally verify the model, we performed a FVD measurement to test the dynamic response characteristic of an oscillator, obtaining the damping coefficient at different amplitude ratios. The experimental setup and corresponding method are described in the “Materials and methods” and Supplementary information sections.

**Fig. 6:**
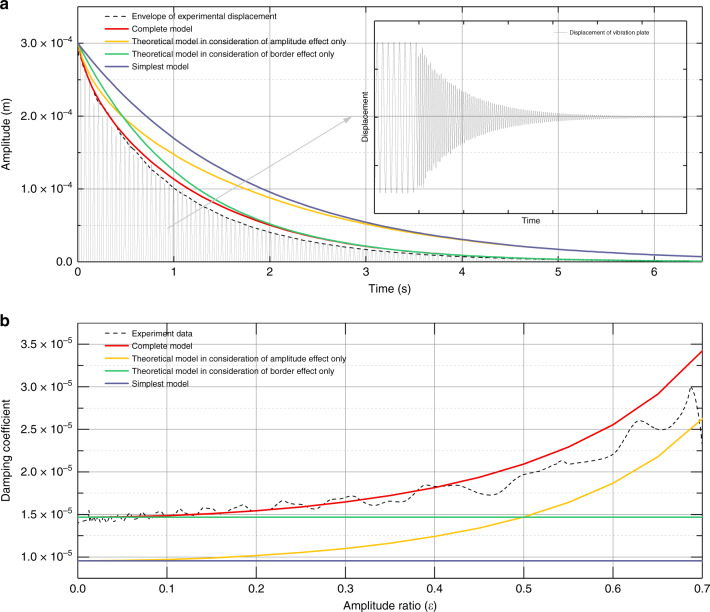
Experimental results. **a** The envelopes of the free-vibration decay response of different theoretical models and experimental data. **b** The damping coefficients obtained from different theoretical models and experimental data.

The FVD response curve of the moving plate is presented by the gray line in Fig. 6a. The vibration of the plate started to decay from the point wherein the amplitude equals 300 μm, and stopped at the point wherein the amplitude is ~0. It should be noted that in different models, the expressions of *c* are totally different, and envelope curves of the FVD of these four models are therefore expressed as:

26 $$\left\{ \begin{array}{ll}d_1 = Ae^{ - \frac{{c_0}}{{2m}}t}\\ d_2 = Ae^{ - \frac{{c_0 \times \left( {1 + \beta } \right)^4}}{{2m}}t}\\ d_3 = Ae^{ - \frac{{c_0}}{{2m\left( {1 - \frac{{x^2}}{{h^2}}} \right)^{1.5}}}t}\\ d_4 = Ae^{ - \frac{{c_0 \times g\left( {\frac{x}{h},\beta ,\gamma } \right)}}{{2m}}t}\end{array} \right..$$

in which *d* is the vibration displacement of an oscillator, *A* is a scale factor involving the initial vibration amplitude, *c*_0_ is the damping coefficient for the simplest model, and *m* is the mass of the moving plate. Substituting the parameters of the tested oscillator, we obtained the calculated envelope curves of the FVD for the four models, as shown as the solid lines in Fig. 6a. It is noted that the best agreement between the result of the complete model and the envelope of the experimental data, depicted by the dashed line, is observed. The result of the model with border effect only matches well with the experiment in the late, while the amplitude is small; the result of the model with amplitude effect only matches well with experiment in the early, while the amplitude is large.

We further calculated the damping coefficient as a function of the amplitude of vibration. The damping coefficient *c* can be obtained through the equation of the envelope curve:

27 $$c = 2m \times \frac{{\mathrm{d}\left( {\ln x} \right)}}{{\mathrm{d}t}}.$$

Figure 6b shows the curves of the damping coefficient versus the amplitude ratio obtained from the theoretical models and experimental data. It is explicitly shown that the curve of the complete model is in best agreement with the experimental result. Similarly, the model with border effects only and the model with amplitude effects only show limited scope of application, whereas they are fit for small- and large-amplitude cases, respectively.

The experimental results, along with the aforementioned comparison, fully verify the accuracy and consistency of our proposed model. The complete model is proven to provide a more trustworthy estimation of the damping coefficient and dynamic response.

#### A quick-calculating factor

**Fig. 7:**
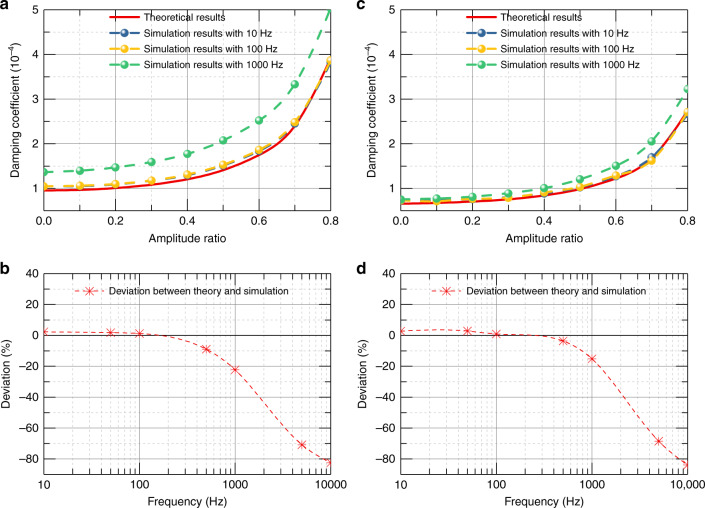
Damping coefficient and deviation with different frequency. **a** Damping coefficient versus amplitude ratio with different frequency for rectangular plate and for **c** circular plate. Deviation of damping coefficient between simulation and theoretical results versus different frequency when the amplitude ratio equals 0.8 for **b** rectangular plate and **d** circular plate.

A series of simulations with different vibration conditions and structures were analyzed to test the validity of our theory, and further develop the complete model and to give a quick-calculating factor of the damping coefficient with the border effect and amplitude effect for various kinds of dimensions. First, simulations with different oscillation frequencies *ω* were performed. Figure 7a, c shows the damping coefficient versus the amplitude ratio with different frequencies for rectangular and circular plates, where the amplitude ratio varies from 0 to 0.8, and the frequency varies from 10 to 1000 Hz. The deviation between the theoretical and simulation results versus frequency when the amplitude ratio is set to 0.8 is depicted in Fig. 7b, d. The results show that the deviation between the theoretical and simulation results is minimal in the frequency range of 10–500 Hz, while it gradually increases >1000 Hz. This is because the squeeze number is large with large frequency so that the fourth term *0*(*σ*^3^) on the right side of Eq. (17) cannot be ignored, which introduces the deviation to the air damping solution.

**Fig. 8:**
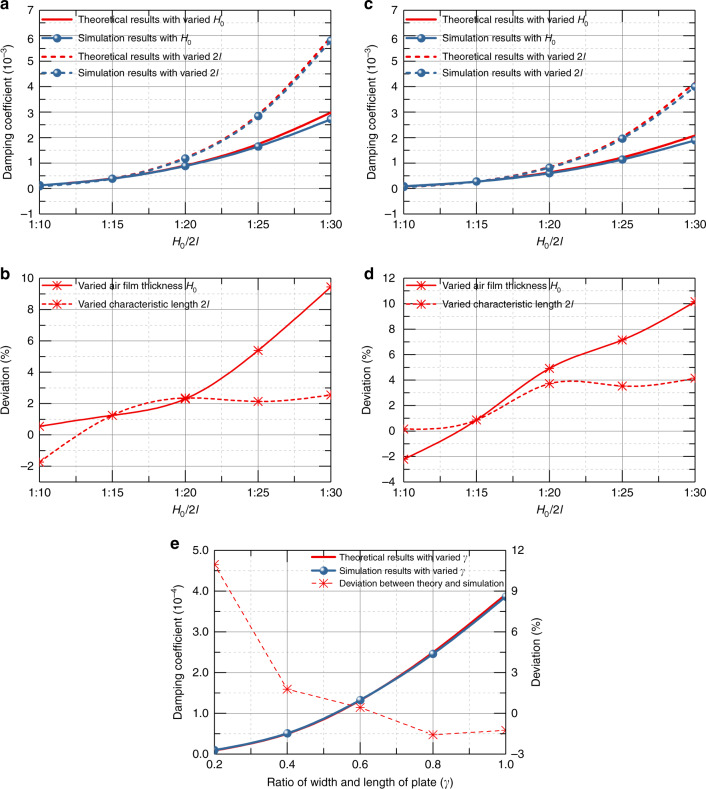
Damping coefficient and deviation with different dimensions. **a**–**b** Damping coefficient and deviation versus different dimensions when amplitude ratio equals 0.8 of rectangular plate. **c**–**d** Damping coefficient and deviation versus different dimensions when amplitude ratio equals 0.8 of circular plate. **e** theoretical and simulated damping coefficient and deviation of rectangular plate versus different length-width ratio when amplitude ratio equals 0.8.

Then, different ratios of *H*_0_*/*2*l* were set, wherein the air film thickness varied from 100 to 300 μm and the characteristic length varied from 1 to 3 mm. The simulation results with an amplitude ratio of 0.8 are compared with theoretical results, as shown in Fig. 8a–d. This indicates that the theoretical deviation can be tolerated when *H*_0_*/*2*l* is in the whole range from 1:10 to 1:30.

Considering different ratios of width and length *γ* for the rectangular plate, more simulations were conducted to investigate the influence of the shape of the rectangular plate. Figure 8e depicts the results of different widths of the rectangular plate, which shows that the rectangular plate shape has little influence on the correctness of the theoretical models.

In summary, all of the simulation results show high consistency with the modified expression Eq. (22) of our proposed complete model for the damping coefficient. Furthermore, the damping coefficient with the border effect, under consideration of the amplitude effect, can be quickly calculated by multiplying the simplest expression Eq. (5) by a factor:

28 $$\begin{array}{lll} g\left( {\varepsilon ,\beta ,\gamma } \right) = \left[ \frac{1}{{\left( {1 - \varepsilon ^2} \right)^{1.5}}} + \left( {3 + \gamma } \right)\beta \frac{{2\left( {1 - \sqrt {1 - \varepsilon ^2} } \right)}}{{\varepsilon ^2\sqrt {1 - \varepsilon ^2} }}\right.\\ \left.+ \,3\left( {1 + \gamma } \right)\beta ^2\frac{{2\left( {1 - \sqrt {1 - \varepsilon ^2} } \right)}{\varepsilon ^2}} + \left( {3\gamma + 1} \right)\beta ^3 + \gamma \beta ^4 \right],\end{array}$$

where *ε* is the amplitude ratio, *γ* is the ratio of the width and length of the plate, and *β* is the factor of the border effect. This factor hybridizes three characteristic values of the border effect, amplitude effect, and shape of the plate to account for all of their influences on squeeze-film air damping. It is a complete theoretical model for the dynamic performance and damping coefficient of vibrated rectangular and circular parallel plates.

## Discussion

Investigation of squeeze-film damping is critical in MEMS design and application, whereas the border effect and amplitude effect have not been considered simultaneously; therefore, large deviations persist when facing complicated boundary conditions and large vibration amplitude cases. This paper combines the merits of the elongation model and amplitude effect function, putting forward a complete model, which gives a complete description of the analytical characterization of the damping performance. The model is thoroughly verified by simulations and experiments, wherein a deviation threefold lower than that of conventional models is obtained by benchmarking. In addition, many simulations with different conditions and geometries of structures are conducted to further extract a quick-calculating factor to modify the damping coefficient. Compared to the time-cost simulation model and design, the complete model and the factor pave the way for a quick and accurate damping design of MEMS devices, which carries great significance for the community.

## Materials and methods

### Numerical analysis

The theoretical calculation was based on the modified Reynolds equation, which stems from the general Reynolds equation^24^, combined with the assumption of an air flow channel^18,19^ and the amplitude effect function^20^.

Numerical analysis based on the finite volume method was used to study the hydromechanical behavior of the oscillating plate. The analysis was performed using ANSYS Fluent commercial software, wherein a series of models were built to characterize different conditions, including the trivial boundary condition and complete boundary condition. As shown in Fig. 2, the volumes represent the fluid, while the cutaway volumes represent the solid volumes. The mesh size around the solid was set to 10 μm, which was much smaller than the dimension of the whole structure. We chose the tetrahedron method of mesh with dynamic mesh setting in the simulation due to the limitation of large amplitude. Ideal compressible air was selected as the material of the fluid in these models. Regarding the trivial boundary conditions, in Fig. 2a, c, four side faces were set to pressure-out boundary conditions, whereas the upper and lower surfaces, which served as the wall, were fixed and controlled to move by program, respectively, similar to Fig. 1c. For the complete models of Fig. 2b, d, six outside surfaces and the inner six surfaces all served as the wall. The latter comprised the moving plate, which was also controlled by the motion program, similar to Fig. 1e. The whole process was conducted by a transient simulation, which included 200 time steps.

Regarding the simulation data analysis, we first obtained the stress by integrating the intensity of pressure on each surface of the moving plate at each time step. The data were then compared with the theoretical results of Eqs. (18) and (19), as performed in Figs. 4 and 5. The squeeze-film air damping can be considered a spring damper, and the load force is expressed as Eq. (20), which indicates that the damping force is the product of the damping coefficient and velocity. The motion of the plate was set to a sinusoidal movement along the designated direction; therefore, the expression of the damping force became the product of the damping coefficient and a cosine function. In this case, the squeeze-film damping coefficient was obtained through triangular Fourier expansion of the simulated force of the moving plate, in which the first-order coefficient was regarded as the damping coefficient and used to compare with the theoretically calculated damping coefficient.

### Experimental analysis

The experiments were carried out in a custom-built vibration test setup, including an oscillator, a standard vibrator, a laser vibrometer, and the corresponding data acquisition system (see Supplementary Information). The time-varying vibration of the oscillator was measured by the vibrometer and high-speed data acquisition system, which enabled the real-time measurement of the FVD response.

The FVD method is based on the FVD response curve, whose displacement with a small damping coefficient is expressed as:

29 $$d = Ae^{ - \frac{c}{{2m}}t}\sin \left( {\omega t + \varphi } \right),$$

in which *d* is the vibration displacement of an oscillator, *A* is a scale factor involving the initial vibration amplitude, *c* is the damping coefficient, *m* is the mass of the moving plate, *ω* is the vibration frequency, and *φ* is the phase. *A*, *ω*, and *φ* can be identified as constant in the case of a small damping ratio; thus, by detecting the decay response, it is able to extract the damping coefficient *c* from the envelope in Eq. (27). Compared to the half-width method^28^, the FVD method^29^ is demonstrated to have higher accuracy for small damping ratios.

## Supplementary information

Supplementary Information for Investigation of complete model of squeeze-film damping of MEMS devices
