# Supplementary material for: Investigation of a complete squeeze-film damping model for MEMS devices
Source: Microsyst Nanoeng. 2021 Jul 22;7:54. doi: 10.1038/s41378-021-00279-6 (PMC8433295; doi:10.1038/s41378-021-00279-6)
Supplement: Supplementary file 1 — Supplementary Information for Investigation of complete model of squeeze-film damping of MEMS devices [file 41378_2021_279_MOESM1_ESM.docx]

**Supplementary Information for**

**Investigation of complete model of squeeze-film damping of MEMS devices**

Qianbo Lu^a,#,*^, Weidong Fang^b,#^, Chen Wang^c, d^, Jian Bai^b,*^, Yuan Yao^e, f^, Jiaxiao Chen^b^, Xiang Xu^b^, Wei Huang^a,*^

^a^ Ningbo Institute of Northwestern Polytechnical University, Frontiers Science Center for Flexible Electronics (FSCFE), MIIT Key Laboratory of Flexible Electronics (KLoFE), Shaanxi Key Laboratory of Flexible Electronics (KLoFE), Institute of Flexible Electronics (IFE), Northwestern Polytechnical University, Xi'an 710072, Shaanxi, China

^b^ College of Optical Science and Engineering, State Key Laboratory of Modern Optical Instrumentation, Zhejiang University, Hangzhou 310027, China

^c^ Department of Electrical Engineering and Computer Science, University of Liege, Liege, Belgium

^d^ ESAT-MNS, University of Leuven, Leuven 3001, Belgium

^e^ Huazhong University of Science and Technology -Wuhan National Laboratory for Optoelectronics, Hubei 430074, China

^f^ Huazhong Institute of Electro-Optics-Wuhan National Lab for Optoelectronics, Hubei 430074, China

^*^Corresponding author: Qianbo Lu ([iamqlu@nwpu.edu.cn](mailto:iamqlu@nwpu.edu.cn)); Jian Bai ([bai@zju.edu.cn](mailto:bai@zju.edu.cn)); Wei Huang ([iamwhuang@nwpu.edu.cn](mailto:iamwhuang@nwpu.edu.cn))

**Supplementary Note 1.**

**Additional information about simulation:**

Regarding the numerical simulation, four models based on Finite Volume Method (FVM) were built to study the hydromechanics behavior of the oscillating plate. Simulation was performed using commercial software, ANSYS Fluent. As sketched in Fig. 2 in the manuscript, Fig. 2 (a) and (c) represent the simplest models (the same as the elongation models) with trivial boundary condition, and Fig. 2 (b) and (d) represent complete models in consideration of border effect for rectangular plate and circular plate, respectively. Note that numerical simulation includes the influence of amplitude effect, so there is no need to set more FVM models to add amplitude effect. Several means were taken to ensure the reliability of our simulation:

1. The mesh size around the plate was set to 10 μm, which is a relatively small value compared to the dimension of whole structure. The thickness of the air film is hundreds of micrometers, the whole model therefore occupies a mesh quantity of more than millions, which enables a trustworthy accuracy of the simulation.

2. We divide one period of the motion to 200 time steps, which is a large enough number, in order to simulate the actual sinusoidal movement correctly.

3. We chose tetrahedrons method of mesh with dynamic mesh setting in the simulation due to the limitation of large-amplitude. Even though hex dominant method of mesh normally has higher accuracy, it is not applicable for the case of large-amplitude. We have performed the comparison between the tetrahedrons method and hex dominant method for the case of small-amplitude, wherein the vibration amplitude was 20 μm. Table S1 indicates that the error of the simulation result utilizing tetrahedrons method is approximately equal to that utilizing hex dominant method, which confirms the validity of our mesh type.

**Table S1 Comparison of simulation results of the tetrahedrons method and hex dominant method for the case of small amplitude**

| Mesh Method | Simulation Results | Theoretical Results |
| --- | --- | --- |
| tetrahedrons method | 7.987×10^-5^ | 7.764×10^-5^ |
| hex dominant method | 7.448×10^-5^ |  |

4. As for the data analysis, what we obtained directly from simulation was the pressure distribution of the surface of the moving plate as a function of time step. Then it was converted to the overall pressure (or force) by area integral, thus, getting the average pressure as a function of time, as depicted in Fig. 4 and Fig. 5 in the manuscript. Referring Eq. (20) and (21), we finally obtained the damping coefficient through time-varying force by following equation:

$c=\sum_{n=1}^{200} \frac{F_{n}Tv_{n}}{\left(\varepsilon h_{0}\omega\right)^{2}}$, (S1)

where *F_n_* is the force applied to the moving plate at *n*th time step, *T* and *ω* are the period and circular frequency of the sinusoidal movement, *v_n_* is the average moving speed of the plate at *n*th time step, *ε* is the amplitude ratio of the plate, *h_0_* is the thickness of the initial air film.

**Supplementary Note 2.**

**Additional information about experiment:**

Supplementary Fig. S1 shows the custom-built vibration test setup, which includes an oscillator, a standard vibrator, a laser vibrometer (Model OFV-503, Polytech Inc.), and corresponding data acquisition system. The oscillator, depicted in the expanded view of Fig. S1, is a three-layer silicon structure, wherein a moving plate with a dimension of 3 mm × 3 mm × 400 μm is suspended in air through four beams, and the thickness of the air film is controlled to 400 μm by attaching an etched silicon die. In addition, the lateral dimension of the air film is set to 8 mm × 8 mm, which is far larger than that of the moving plate. An accelerometer (Model 8305, Brüel & Kjær Inc.) was implemented to monitor the vibration of the vibrator, so that we could obtain the input to the oscillator. The temperature and humidity were adjusted to 22℃ and 42%, respectively, corresponding to the coefficient of viscosity of 1.8185×10^-5^, which is identical to our used number in the calculation.


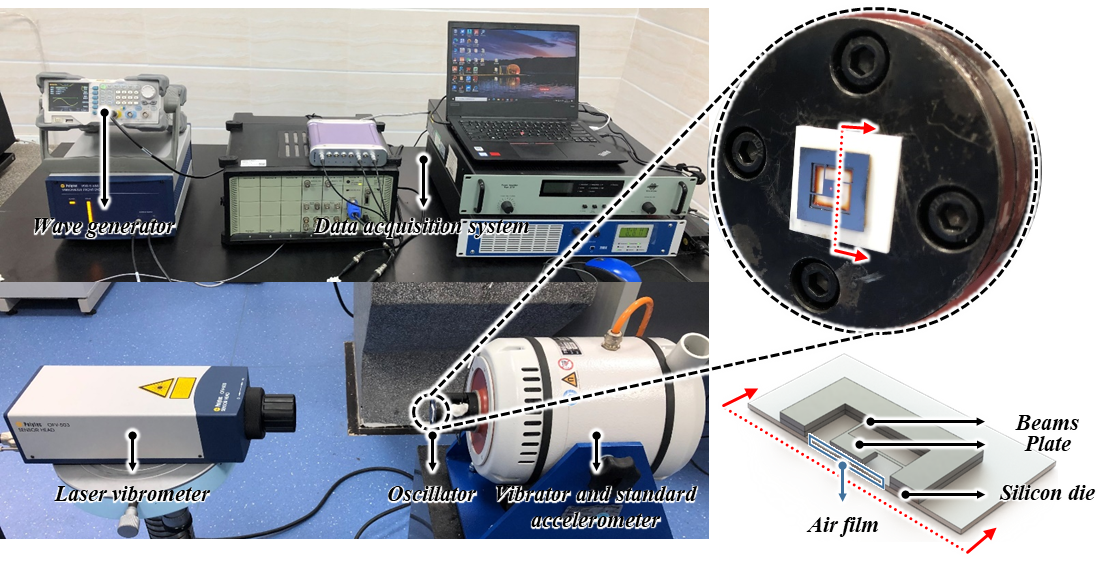


Fig. S1 The experimental setup and the tested structure.

Compared to half-width method^1^, the free-vibration decay (FVD) method^2^ is demonstrated to have higher accuracy for small damping ratio. FVD method is based on the free-vibration decay response curve, whose displacement with small damping coefficient is expressed as:

, (S2)

in which *d* is the vibration displacement of an oscillator, *A* is a scale factor involving the initial vibration amplitude, *c* is the damping coefficient, *m* is the mass of moving plate, *ω* is the vibration frequency, *ϕ* is the phase. *A*, *ω*, *ϕ* can be identified as constant in the case of small damping ratio, thus by detecting the decay response, it is able to extract the damping coefficient *c*.





Fig. S2 Damped free-vibration decay response curve of the moving plate.

Herein, we utilized FVD method to obtain the specific damping coefficient. The free-vibration decay response curve of the moving plate is presented in Fig. S2. The vibration of the plate started to decay from the point where the amplitude equals 300 μm, and stopped at the point where the amplitude is approximate to 0. It should be pointed out that in different models, the expressions of *c* are totally different, while in the simplest model *c* is a constant *c*_0_, in the model with border effect only *c* is related to the factor of border effect *β*, in the model with amplitude effect only *c* is related to the vibration amplitude, in the complete model *c* should be multiplied by *g*(*ε*,*β*,*γ*). The envelop curves of the free-vibration decay of these four models are therefore expressed as:

$\left\{\begin{matrix} d_{1}=Ae^{-\frac{c_{0}}{2m}t} \\ d_{2}=Ae^{-\frac{c_{0}\times\left(1+\beta\right)^{4}}{2m}t} \\ d_{3}=Ae^{-\frac{c_{0}}{2m\left(1-\frac{x^{2}}{h^{2}}\right)^{1.5}}t} \\ d_{4}=Ae^{-\frac{c_{0}\times g\left(\frac{x}{h},\beta,\gamma\right)}{2m}t} \end{matrix}\right.$. (S3)

Substituting parameters of the tested oscillator, the envelop curves of the free-vibration decay for four models were obtained, as shown as the solid lines in Fig. (6) in the manuscript.

In order to validate the effectiveness more intuitively, we further calculated the damping coefficient, as a function of the amplitude of vibration. The damping coefficient *c* can be obtained through the equation of the envelope curve:

$c=2m\times\frac{d\left(\ln x\right)}{dt}$. (S4)

**Supplementary References**

1 Pandey, A.K. & Pratap, R. A comparative study of analytical squeeze film damping models in rigid rectangular perforated MEMS structures with experimental results. *Microfluidics and Nanofluidics*, **4**, 205-218, (2008).

2 Senetakis, K., Anastasiadis, A. & Pitilakis, K. A comparison of material damping measurements in resonant column using the steady-state and free-vibration decay methods. *Soil Dynamics and Earthquake Engineering*, **74**, 10-13, (2015).
